# Reply: Managing Atypical Fungal Endocarditis: Better Be Prepared Than Improvize?

**DOI:** 10.1016/j.jaccas.2025.106307

**Published:** 2025-12-17

**Authors:** Genichiro Fujioka, Jacob A. Powell, Michael Tang

**Affiliations:** a Texas A&M College of Medicine, Bryan, Texas, USA; b Baylor University Medical Center, Dallas, Texas, USA

Thank you Drs Khlidj and Ahmed for your interest and bringing up important points to discuss about our paper.^1^

Drs Khlidj and Ahmed propose that transthoracic echocardiography (TTE) should be used in all patients with fungemia to rule out vegetation like in our patient without common risk factors. While European guidelines recommend a screening TTE in patients with candidemia, the more recent Infectious Disease Society of America guidelines do not recommend this practice as the prevalence of candida endocarditis in patients with candidemia is as low as 1.9%.^2^ However, these same guidelines suggest a TTE is warranted when blood cultures are persistently positive, patients have fever despite treatment, and there are new heart failure symptoms, embolism, or a new murmur. A TTE should also be done in higher-risk individuals with risk factors for candida endocarditis.

The letter also suggests that the initial antifungal therapy failure was a result of multidrug resistance. Although we do not have sensitivities for the *Candida parapsilosis* found in our patient, it was unlikely that drug resistance played a significant role in treatment resistance. Treating *C. parapsilosis* with echinocandins typically requires a higher minimal inhibitory concentration than that for other *Candida* species, but resistance to polyenes is rare.^3^ It is possible that the *C. parapsilosis* found in our patient was resistant to micafungin and/or liposomal amphotericin B, but it would be exceedingly rare and would not explain the effectiveness of combined therapy. Rather, it is more likely that combination therapy was more effective at treating resistance due to biofilm production.^4^

Due to the large size of vegetation and the persistent fungemia, early surgical intervention would have been preferred over antifungal therapy alone.^5^ However, perioperative optimization of the patient is still critical in decreasing morbidity and mortality from cardiac surgery.
